# Exploring racial disparities in treatment patterns and outcomes for patients with multiple myeloma using real world data

**DOI:** 10.1038/s41408-022-00665-x

**Published:** 2022-04-19

**Authors:** Kathleen Maignan, Lola A. Fashoyin-Aje, Aracelis Z. Torres, Laura L. Fernandes, Thomas Gwise, Shrujal B. Baxi, James P. Roose, Donna R. Rivera, Yuan Li Shen, Paul G. Kluetz, Nicole J. Gormley

**Affiliations:** 1 Flatiron Health Inc, New York, NY USA; 2 Center for Drug Evaluation and Research, US Food and Drug Administration, Silver Spring, MD USA; 3 Oncology Center of Excellence, US Food and Drug Administration, Silver Spring, MD USA

**Keywords:** Epidemiology, Myeloma

## Abstract

This retrospective observational study evaluated racial disparities among Black and White patients with multiple myeloma (MM). We included patients from a longitudinal de-identified EHR-derived database who had ≥2 visits recorded on or after 1/1/2011, documented treatment, and race listed as White or Black. Black patients (*n* = 1172) were more likely female (54.8%/42.9%) and younger (<65 years, 40.8%/30.8%) than White patients (*n* = 4637). Unadjusted median real-world overall survival (rwOS) indexed to first-line of therapy (LOT) was 64.6 months (95% CI: 57.8–74.0) for Blacks and 54.5 months (95% CI: 50.9–56.2) for Whites. Adjusted rwOS estimates (for sex, age at index date, and practice type) to either first- (aHR = 0.94; 95% CI: 0.84–1.06) or second-LOT (aHR = 0.90; 95% CI: 0.77–1.05) were similar. Unadjusted derived response rate (dRR) during first-LOT was 84.8% (95% CI: 80.7–88.1) for Blacks and 86.9% (95% CI: 85.0–88.5) for Whites (odds ratio [OR] = 0.78 [95% CI: 0.57–1.10]); in second-LOT, 67.2% (95% CI: 58.4–75.0) for Blacks and 72.4% (95% CI: 68.1–76.3) for Whites (OR = 0.72 [95% CI: 0.46–1.13]). High representation of Black patients enabled this robust analysis, albeit with limitations inherent to the observational data source, the retrospective design, and the analytic use of newly derived endpoints requiring further validation.

## Introduction

Multiple myeloma (MM) is a hematologic cancer caused by the proliferation of malignant plasma cells in the bone marrow. The National Cancer Institute Surveillance Epidemiology and End Results (SEER) database estimates 35,000 new cases of MM and 12,000 deaths attributable to MM in 2021 in the United States [1]. The incidence rate among African Americans is more than double than in Whites. Age-adjusted incidence and death rates in African Americans compared with Whites are 14.3 vs. 6.1 cases per 100,000 and 6.0 vs. 2.9 cases per 100,000. Various studies have suggested potential differences in the underlying disease biology based on race. African Americans develop MM at a younger age than Whites, their tumors are more likely to have translocations involving chromosome 14 and the immunoglobulin heavy chain (IgH) gene, and less likely to harbor *TP53* mutations [2–4].

Even when adjusted by incidence, the mortality risk due to MM appears higher among African Americans in the United States. Studies evaluating overall survival (OS) have shown either similar, or in some cases, better survival for African Americans compared to Whites when there was equal access to care [5, 6]. To date, many of the analyses assessing race-based outcomes in MM have relied on SEER-based data or on single-institutions or trial-based results. To our knowledge, electronic health record (EHR)-derived real-world data (RWD) sources have not previously been utilized to assess outcomes in MM based on race. We describe an analysis comparing outcomes such as response rate and OS using EHR-derived data in a cohort of Black and White patients.

## Methods

### Data source

This retrospective observational study used data from a longitudinal, de-identified EHR-derived Flatiron Health database, which includes patient-level structured and unstructured data curated via technology-enabled abstraction [7, 8]. As of the end of the observation period (May 31, 2019), the database consisted of ~280 US cancer clinics (~800 sites of care).

The study population included patients diagnosed with MM selected by presence of a structured diagnosis code (ICD-9 203.x or ICD-10 C90.0x, C90), and with at least two visits on or after January 1, 2011. A physician statement confirming the MM diagnosis on or after January 1, 2011 was required (via abstraction of unstructured data). Patients were required to have received at least one line of therapy (LOT) evidenced by a structured non-canceled medication order of intravenous (IV) therapy, a structured medication administration of IV therapy, or abstracted information on oral therapy for lenalidomide, thalidomide, pomalidomide, cyclophosphamide, dexamethasone, panobinostat, ixazomib, melphalan, study drugs, and prednisone, as well as a race category listed as either White or Black in the EHR. Patients were excluded if the abstracted start date of MM treatment was more than 30 days before the start of structured EHR activity.

Institutional Review Board approval of the study protocol for data collection was obtained prior to study conduct, and included a waiver of informed consent.

### Endpoints

Real-world overall survival (rwOS) was defined as time from index date (defined as the start date of the first-line or second-line therapy) to the date of death. For censoring, the last confirmed activity date (i.e., last structured visit or last oral cancer drug episode from unstructured data) was used. Dates of death were based on a composite mortality variable [9].

Laboratory data were used to derive disease burden according to the adaptive version of International Myeloma Working Group (IMWG) criteria. Several IMWG criteria (immunofixation, bone marrow plasma cell percentage, imaging [bone lesions, soft tissue plasmacytomas], and hypercalcemia) were not factored into deriving disease burden due to inconsistent collection of these data in routine care. Serum protein electrophoresis (SPEP) and 24-h urine protein electrophoresis (UPEP) data were abstracted from unstructured health record notes while structured laboratory information was used to identify free light chain (FLC) data.

For each patient and LOT, a single specimen type (i.e., serum or urine) was selected as the primary specimen type for establishing a baseline M-spike value and for subsequent follow up assessments, using a hierarchy that incorporates test type and availability of measurable results using standard units from 90-days prior to the LOT start date through the end of the patient record; SPEP tests were preferentially selected if a record of ≥1 g/dl was recorded; otherwise, UPEP tests were selected if a record of ≥200 mg/24 h was recorded. In the absence of M-spike information, FLC was selected if the following conditions were met: FLC values were reported in mg/dl, and both kappa and lambda were tested on the same day.

A derived real world response rate (dRR) was calculated as the proportion of patients meeting the threshold for a derived partial response (dPR) or derived very good partial response (dVGPR) among all patients with a qualifying baseline lab and at least one subsequent lab during the first- or second-LOT regimens of interest. Analogs for complete response (CR) and stringent CR were not derived because bone marrow biopsy results were not routinely available. The highest lab value of the selected specimen type among labs occurring within 90 days (before or after) of the LOT start date was initially selected as baseline [10]. However, only patients whose baseline lab was identified as occurring within 30 days prior to and including the LOT start date were included in the primary analysis of derived response in order to improve the clinical relevance of the baseline. As disease burden was assessed over time, subsequent labs were required to have the same specimen type and units and occur at least 14 days after the baseline laboratory assessment. After 90 days, the initial baseline was reset in the event of an increase in the lab value from the baseline or the start of a new LOT. For dPR and dVGPR, events were assessed relative to the baseline specific to the first- or second-LOT regimens of interest. For patients followed using SPEP, dPR was defined as a ≥ 50% reduction in M-spike from baseline, and a dVGPR as M-spike not detectable on electrophoresis. For patients followed using UPEP, dPR was defined as a ≥ 90% reduction in M-spike from baseline or a reduction to < 200 mg/24 h, and a dVGPR as M-spike not detectable on electrophoresis. For patients followed using FLC, a dPR was defined as a ≥ 50% decrease in the difference between involved and uninvolved FLC levels.

### Statistical analysis

Descriptive statistics of baseline demographics, clinical characteristics, and longitudinal MM treatment patterns for the cohort were calculated by race. For de-identification reasons, age was capped at 85 in all analyses. The distributions of rwOS were estimated using the Kaplan–Meier method. A multivariable Cox proportional hazards regression model was used to estimate an adjusted hazard ratio (aHR) after accounting for sex and age at the index date, and for practice type. The covariates were selected based on clinical relevance and completeness at baseline. Among patients with a baseline lab and at least one post-baseline lab during first- or second-LOT, the proportion of patients with dPR or better was reported for each LOT separately. A multivariable logistic regression model adjusted for sex, age at the index date, and practice type (i.e., academic or community) was used to estimate adjusted odds ratios (aORs). Analyses indexed to the start of first- or second-LOT, were presented separately. There were no adjustments for multiplicity in the various comparisons, the nominal 5% level of significance was used to construct the 95% confidence intervals and hence no p-values are reported. All statistical analyses were performed using R, version 3.6.1 (2019-07-05).

## Results

A total of 5809 patients with eligible treatments were included in the overall cohort (Supplementary Fig. 1 and Table 1). The cohort consisted of 20.2% (*n* = 1172) Black patients and 79.8% (*n* = 4637) White patients. Black patients were more likely to be female (54.8% vs. 42.9%), younger at the start of first-LOT (40.8% vs. 30.8% were <65 years old), and from the South US region (61.3% vs. 34.1%) as compared to White patients. While renal and hepatic function derived from structured laboratory data did not differ between Black and White patients, missingness ranged from 25.8% to 63.5% across reported labs (i.e., hemoglobin, platelet count, absolute neutrophil count, creatinine clearance, hepatic-related tests) (Supplementary Table 1). Similar trends in laboratory result availability were seen among the patients with evidence of receiving a second-LOT and among patients included in dRR analyses indexed to first- and second-LOT (Table 1 and Supplementary Tables 1 and 2). During the overall study period, first-LOT distributions were similar for Black and White patients; bortezomib, dexamethasone, and lenalidomide combination regimen comprised the highest proportion for Black (34.7%) and White (33.8%) patients.

**Table 1:** Study population.

Patient characteristic	rwOS population, *n* (%) *N* = 5809		dRR population, *n* (%) *N* = 1824	
	Black (*n* = 1172)	White (*n* = 4637)	Black (*n* = 362)	White (*n* = 1462)
Sex				
Female	642 (54.8)	1989 (42.9)	195 (53.9)	621 (42.5)
Male	530 (45.2)	2648 (57.1)	167 (46.1)	841 (57.5)
Practice type				
Academic	93 (7.9)	555 (12.0)	29 (8.0)	175 (12.0)
Community	1079 (92.1)	4082 (88.0)	333 (92.0)	1287 (88.0)
Age at 1L start				
Median [IQR], years	67.0 [59.0;75.0]	71.0 [62.0;78.0]	68.0 [60.0;75.0]	70.0 [62.0;77.0]
<65 years	478 (40.8)	1426 (30.8)	139 (38.4)	455 (31.1)
65–74 years	391 (33.4)	1507 (32.5)	129 (35.6)	494 (33.8)
75+ years	303 (25.9)	1704 (36.7)	94 (26.0)	513 (35.1)
Region^a^				
Midwest	120 (10.2)	742 (16.0)	37 (10.2)	231 (15.8)
Northeast	178 (15.2)	976 (21.0)	44 (12.2)	272 (18.6)
South	719 (61.3)	1580 (34.1)	240 (66.3)	579 (39.6)
West	46 (3.9)	695 (15.0)	11 (3.0)	196 (13.4)
Other/unknown	109 (9.3)	644 (13.9)	30 (8.3)	184 (12.6)
ECOG PS at 1L start^b^				
0–1	364 (31.1)	1539 (33.2)	105 (29.0)	539 (36.9)
>1	132 (11.3)	433 (9.3)	45 (12.4)	167 (11.4)
Unknown	676 (57.7)	2665 (57.5)	212 (58.6)	756 (51.7)
ISS stage				
Stage I	244 (20.8)	812 (17.5)	73 (20.2)	287 (19.6)
Stage II	202 (17.2)	887 (19.1)	79 (21.8)	328 (22.4)
Stage III	181 (15.4)	883 (19.0)	63 (17.4)	330 (22.6)
Unkn/not docum.	545 (46.5)	2055 (44.3)	147 (40.6)	517 (35.4)
Year of MM diagnosis				
2011–2013	340 (29.0)	1323 (28.5)	99 (27.3)	366 (25.0)
2014–2016	471 (40.2)	1954 (42.1)	149 (41.2)	615 (42.1)
2017–2019	361 (30.8)	1360 (29.3)	114 (31.5)	481 (32.9)
Most common first LOT				
Bortez, Dex, Len	407 (34.7)	1568 (33.8)	161 (44.5)	625 (42.7)
Dex, Len	212 (18.1)	798 (17.2)	54 (14.9)	232 (15.9)
Bortezomib, Dex	168 (14.3)	681 (14.7)	45 (12.4)	187 (12.8)
Bortez, Cyc, Dex	129 (11.0)	512 (11.0)	45 (12.4)	154 (10.5)
Dex	67 (5.7)	237 (5.1)	21 (5.8)	47 (3.2)
Other 1L	189 (16.1)	841 (18.1)	36 (9.9)	217 (14.8)
Specimen type for 1L response				
FLC	NA	NA	51 (14.1)	183 (12.5)
SPEP	NA	NA	303 (83.7)	1237 (84.6)
UPEP	NA	NA	8 (2.2)	42 (2.9)
Stem Cell Transplant in 1L	184 (15.7)	910 (19.6)	60 (16.6)	343 (23.5)
1L Maintenance Therapy	216 (18.4)	916 (19.8)	79 (21.8)	324 (22.2)
Median follow-up time from 1L start date [IQR], months^c^	24.1 [9.9;44.8]	23.7 [9.3;43.0]	26.0 [12.4;45.1]	24.6 [10.3;43.4]
Median time from MM Diagnosis to 1L start [IQR], days	29.0 [17.0;46.2]	30.0 [18.0;45.0]	28.0 [17.0;41.0]	28.0 [18.0;40.0]

Baseline characteristics of cohort indexed to start of first-LOT by race and rwOS/dRR population.

*dRR* derived response rate, ECOG PS Eastern Cooperative Oncology Group performance score, *IQR* interquartile range, *ISS* International Staging System, *LOT* line of therapy, *MM* multiple myeloma, *rwOS* real-world overall survival.

^a^Regions are based on the United States census region of the patient’s state of residence. Region is reported as Other/Unknown for patients from academic sites for de-identification reasons.

^b^ECOG is determined using records from 30 days prior to and up to 7 days after the 1L start date. If there are multiple ECOG values at the same absolute distance from the 1L start date, priority is given to the ECOG value that precedes the index date. For patients with multiple ECOG values recorded on the same day, the highest value is used.

^c^Follow-up time is defined as time from the start date of first-line therapy to either date of death, if known, or the patient’s last confirmed activity (i.e., clinic visit or abstracted oral drug episode).

**Fig. 1:**
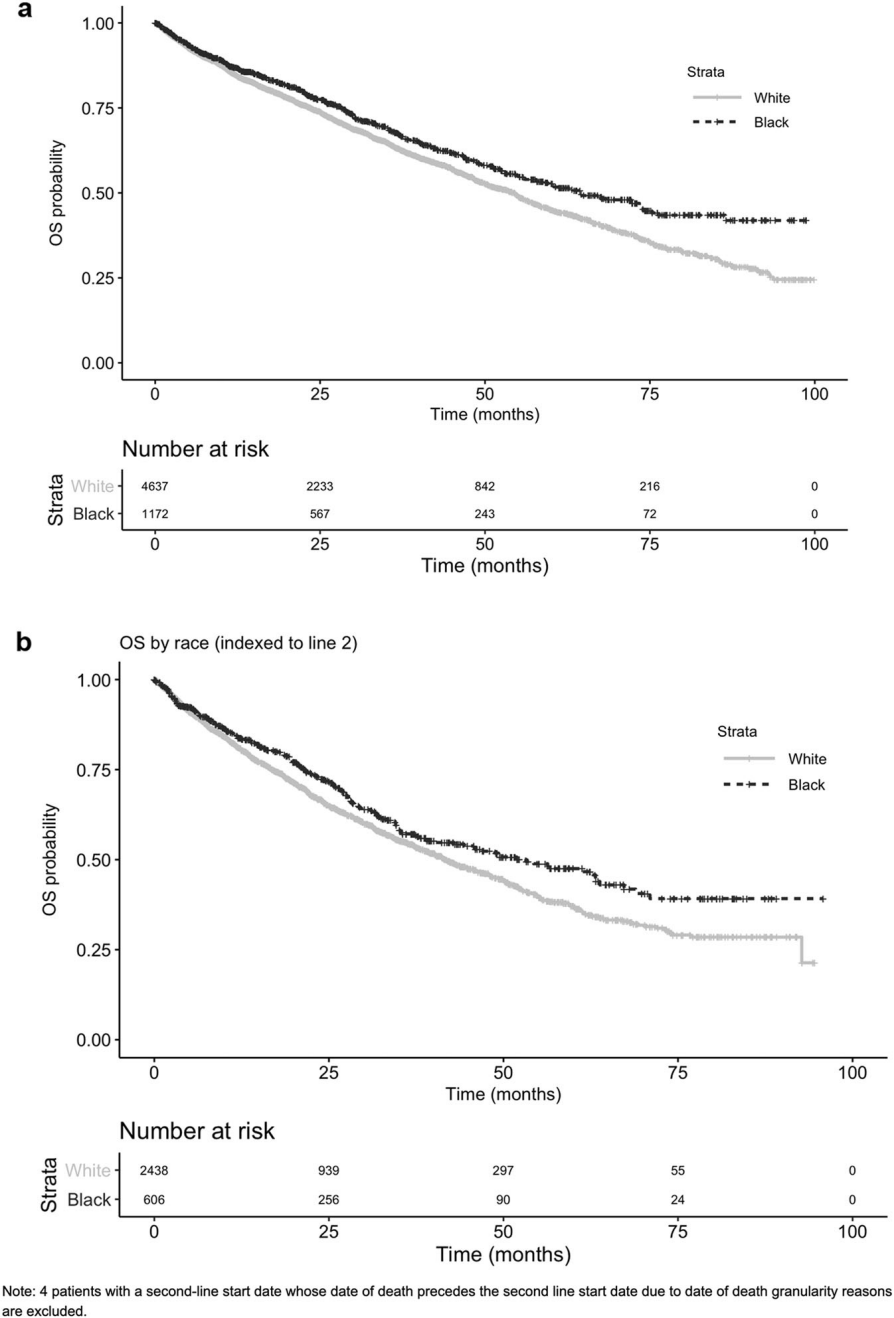
Kaplan–Meier estimates of rwOS by race. **a** rwOS indexed to first line of therapy. **b** rwOS indexed to second line of therapy.

In unadjusted rwOS analyses indexed to first-LOT, Black patients (*n* = 1172) had longer survival with a median of 64.6 months (95% CI: 57.8, 74.0) compared to 54.5 months (95% CI: 50.9, 56.2) for White patients (*n* = 4637) (Fig. 1a). When indexing to second-LOT, the unadjusted median estimate was also longer for Black patients (*n* = 606) at 53.2 months (95% CI: 40.1, 63.8) vs. 41.7 months (95% CI: 38.7, 45.7) for White patients (*n* = 2442) (Fig. 1b). When adjusted by sex, age at index, and practice type, rwOS differences by race were no longer observed when anchored to first-LOT start, (aHR 0.94; 95% CI: 0.84, 1.06) and when indexed to second-LOT start (aHR 0.90; 95% CI: 0.77, 1.05) (Table 2). rwOS by the most common (i.e., at least 10% of study population) specific first-LOT regimens showed similar associations (Supplementary Table 3a). Sensitivity rwOS analyses within the subset of patients eligible for the response analyses at the first-LOT and second-LOT produced similar results.

**Table 2:** Real-world overall survival analysis.

	rwOS aHR (95% CI)	Events	Median rwOS (95% CI), months
First LOT (*N* = 5809)			
Black (*N* = 1172)	0.94 (0.84, 1.06)	371	64.6 (57.8, 74.0)
White (*N* = 4637)	Ref.	1719	54.5 (50.9, 56.2)
Second LOT (*N* = 3048)			
Black (*N* = 606)	0.90 (0.77, 1.05)	209	53.2 (40.1, 63.8)
White (*N* = 2442)	Ref.	963	41.7 (38.7, 45.7)

White patients are the reference group. Relative hazard of death from start of first line and second line treatment between Blacks and Whites, adjusting for sex, age, and practice type.

*aHR* adjusted hazard ratio, *CI* confidence interval, *LOT* line of therapy, *MM* multiple myeloma, *rwOS* real-world overall survival.

Within the overall study population, there were 31.5% (*n* = 1462) of White patients and 30.9% (*n* = 362) of Black patients with two non-null labs that qualified them for inclusion in the response analyses for their first-LOT. Among these eligible patients, the unadjusted dRR was 86.9% (95% CI: 85.0%, 88.5%) for White patients and 84.8% (95% CI: 80.7%, 88.1%) for Black patients. Among patients with a second-LOT, 18.6% (*n* = 453) of White patients and 19.6% (*n* = 119) of Black patients were assessable for dRR, and the unadjusted dRR was 72.4% (95% CI: 68.1%, 76.3%) and 67.2% (95% CI: 58.4%, 75.0%) for White patients and Black patients, respectively. Upon adjusting for age, sex, and practice type, the dRR in first-LOT remained similar when comparing Black patients to the reference group of White patients with an aOR of 0.78 (95% CI: 0.57, 1.10). Likewise, the relative odds of responding during the second-LOT for Black patients relative to White patients was 0.72 (95% CI: 0.46, 1.13) (Table 3).

**Table 3:** Response analysis.

	dRR odds ratio (95% CI)	Responders	dRR (95% CI)
First LOT (*N* = 1824)			
Black (*N* = 362)	0.78 (0.57, 1.10)	307	84.8% (80.7%, 88.1%)
White (*N* = 1462)	Ref.	1270	86.9% (85.0%, 88.5%)
Second LOT (*N* = 572)			
Black (*N* = 119)	0.72 (0.46, 1.13)	80	67.2% (58.4%, 75.0%)
White (*N* = 453)	Ref.	328	72.4% (68.1%, 76.3%)

White patients are the reference group. Relative odds of responding to first line and second line treatment between Black Patients and White Patients, adjusting for sex, age, and practice type.

*dRR* derived response rate, *LOT* line of therapy.

## Discussion

Previous analyses stratified by or adjusting for race have demonstrated similar or possibly even improved survival outcomes between Black and White patients with MM. This study demonstrated an opportunity for evaluating real world outcomes with retrospective EHR-derived data, by investigating response outcomes not routinely evaluable, and complementing insights from prospective trials beleaguered by relatively limited representativeness and the challenges associated with sub-analyses [11].

This study population consisted of 20% Black patients, adequately representing the disease epidemiology and the incidence of the disease in Black patients [12–14]. The adjusted analysis demonstrated similar OS results for Black and White patients, although the unadjusted analyses suggested a trend toward improved survival in Black patients. These findings are consistent with previous analyses suggesting that younger age at diagnosis and different cytogenetic profile may contribute to similar or more favorable survival outcomes in Black patients. Most recently, results from the CoMMpass registry showed superior survival for White patients when compared to Black patients, a difference seemingly driven, to considerable extent, by different patterns of care (with less utilization of triplet therapy and ASCT in Black patients) [15], as also suggested by a recent SEER-based report [16]. Of note, our study was US-based while the CoMMpass registry is worldwide, which may explain the overall differences in care (ASCT being a more common practice outside of the US [17, 18]) and diverging outcomes. These studies highlight the urgency to conduct more inclusive studies to better understand the potential interactions between clinical treatment patterns, epidemiologic trends, and access to care.

An additional strength of this study was the derivation of a response endpoint using a RWD source where lab-based information was captured. The dRR analyses showed no differences, but their overall smaller sample sizes make interpretation challenging. Furthermore, the requirement for baseline labs for the response derivation to happen within the 30-day window prior to the start of each LOT could have introduced selection bias.

This study reflects real world treatment patterns (i.e., proteasome inhibitor and immunomodulatory agent use) among Black and White patients with MM. RWD may provide insights on the translation of RCT evidence into standard practice and may be hypothesis-generating about potential effectiveness in populations more diverse than clinical trials, for instance, in patients ineligible or not enrolled in clinical trials for a variety of reasons (e.g., access, structural, or sociological barriers). In an FDA review of new MM drug applications submitted for approval between 2003–2017, only 4.5% of patients were Black [19]. RWD, as a complement to clinical trial data, could improve generalizability.

This study has several limitations, including the potential for selection bias due to the inherent nature of observational studies, and driven by study inclusion criteria. Considering the full originating database, the cohort used for the rwOS analysis included 44% of patients with an MM diagnosis, and the dRR analysis included 14% (Supplementary Fig. 1). The sample size of the Black cohort remained limited, particularly for the analyses of dRR and treatment-defined subgroups. Thus, the resulting populations may not be representative of patients seen across U.S practices or care settings, which ultimately may limit the generalizability of results.

The accuracy of race as captured in RWD sources may vary, and can be clinician-reported or patient self-reported, which has been shown to increase accuracy [20]. In this study, race was a self-reported variable; while inconsistency in documentation practices may have impacted completeness and accuracy, patient racial distribution may reflect the real world setting more accurately than clinical trials.

Currently, no consensus exists on the clinical utility and adequacy of certain RWD endpoints (i.e., dRR and rwOS), as they inherently differ from clinical trial endpoints considered the gold standard in evaluating outcomes for drug approval, and the association between the two is unclear. Potential gaps include: (1) RWD are not collected systematically and patients may have extended periods without assessments or may not have adequate follow-up time; and (2) laboratory assessments and response criteria may differ by physician or practice settings. In this study, absent laboratory values may indicate the following: not performed (referred to as “no or not conducted”), occurring outside of the data collection setting, unverified, or not mapped to the research database (referred to as “unknown” or “missing”). The ability to distinguish between these categories is marginal and their impact should be minimized for optimal analysis interpretation, as they could lead to misclassification or confounding of dRR results.

As stated in the methods, we evaluated response using several IMWG criteria adaptations to account for the non-systematic nature of RWD collection. Altogether, these adaptations could have introduced selection bias based on data collection and availability. Furthermore, the adequacy of this “adapted” algorithm for response derivation warrants further evaluation. For instance, inclusion required patients to have a baseline lab for the response algorithm within the 30-day window prior to LOT initiation. Responses of “PR or better” did not have CR assessments, and only one specimen type was used for each patient. Some responders’ M-Spike or FLC labs may have initially increased after therapy initiation and before decreasing into a response event. When deriving time-to-event endpoints using RWD (i.e., rwOS), the concern for immortal time bias with respect to the time from diagnosis to the index date, the differential follow-up and other unobserved selection biases (such as those potentially associated to the requirement of having received therapy) should be considered in the interpretation of results. Even without differences in diagnostic time to index date and follow-up duration (see Table 1), differences in survival between diagnosis and index date and other unobservable confounding factors cannot be ruled out.

This analysis compares two populations derived from the same database; however, without randomization, both observable and unobservable factors could differentially impact the two populations and introduce bias. The observable factors may include practice settings, regions and the prognostic factors evaluable in the dataset. Unobservable factors may include cytogenetics and molecular markers, social economic status, or other factors not available or unlikely to be captured in observational data sources, such as patient and clinician preferences. While statistical methods can be used to adjust for known observed confounders, those confounders may also have high missingness levels which may limit interpretability. Additionally, the benefit of including these factors in the analysis must be balanced with the desire for robust cohort sizes. Inclusion of more prognostic factors would likely reduce the size of an analytic cohort restricted to patients with complete data, while other methods to account for missing data may require additional assumptions.

Our analysis provides a robust assessment of treatment patterns and outcomes in Black patients, historically under-represented in MM clinical trials compared to White patients. Efforts should continue to improve representation in clinical trials. This analysis serves as an example of how RWD can be used to complement our knowledge of treatment outcomes across diverse patient populations. Development of methodological and analytical strategies that account for the inherent limitations of RWD while utilizing its detailed longitudinal structure will enhance the ability to glean insights from this data source and lead to a more comprehensive understanding of real-world clinical practices and outcomes.

## Supplementary information

Supplemental Material

## Data Availability

The data that support the findings of this study have been originated by Flatiron Health, Inc. These de-identified data may be made available upon request, and are subject to a license agreement with Flatiron Health; interested researchers should contact <DataAccess@flatiron.com> to determine licensing terms.
