# Supplementary material for: Exploring racial disparities in treatment patterns and outcomes for patients with multiple myeloma using real world data
Source: Blood Cancer J. 2022 Apr 19;12(4):65. doi: 10.1038/s41408-022-00665-x (PMC9018767; doi:10.1038/s41408-022-00665-x)

**Exploring Racial Disparities in Treatment Patterns and Outcomes Among Patients with Multiple Myeloma using Real World Data**
Kathleen Maignan; Lola A. Fashoyin-Aje; Aracelis Z. Torres, PhD; Laura L. Fernandes; Thomas Gwise; Shrujal B. Baxi, MD MPH; James P. Roose; Donna R. Rivera; Yuan Li Shen; Paul G. Kluetz; Nicole J. Gormley.

##

## **SUPPLEMENT**

###

### **Methods**

**Line of Therapy Rules Derivation**

Flatiron Health’s line of therapy (LOT) business rules are disease-specific rules that summarize the sequence of antineoplastic, systemic therapies in the cancer setting of interest. Flatiron applies rules to drug tables (containing drug orders, administrations, and abstracted oral therapy data), and disease-specific data (e.g., dates of bone marrow transplants identified in patient charts) to derive disease-specific lines of therapy.

In multiple myeloma, the LOT rules identify the first LOT based on the first drugs received after or up to 14 days before the patient’s multiple myeloma diagnosis date. A LOT is defined as the first eligible drug (an antineoplastic or steroid), plus other eligible drugs given within 28 days. Drugs given as maintenance therapy (potentially bortezomib, lenalidomide, thalidomide, or ixazomib) do not advance the line number, while initiation of new treatments or a gap in treatment of more than 90 days will advance the line number (with some exceptions, e.g., substitution of daratumumab and daratumumab/hyaluronidase or reference product for a biosimilar). Transplants that occur during a line do not advance the line number but are reflected in LOT data.

**Derived Response Baseline Lab and Patient Inclusion**

**
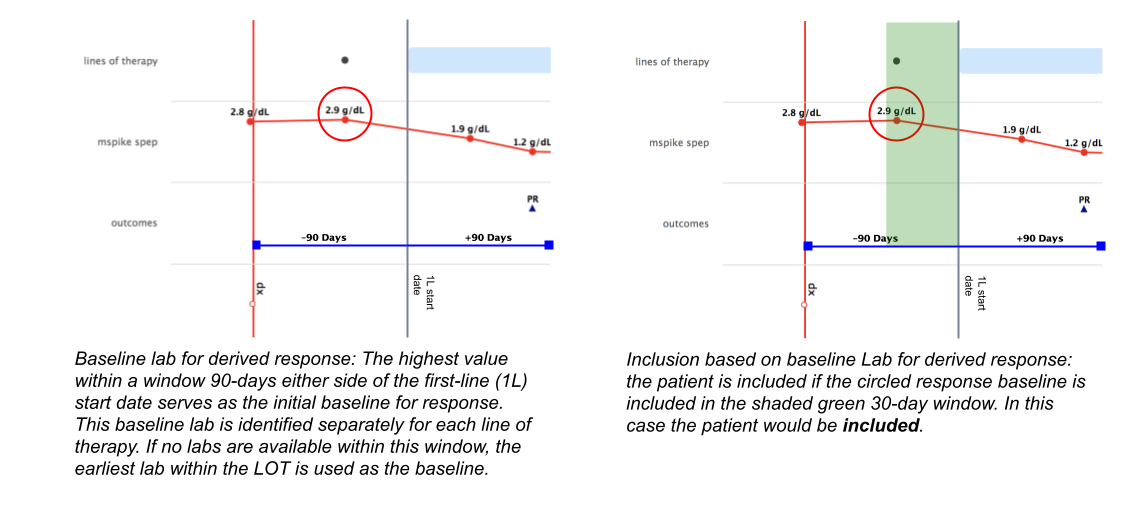
**

### **Supplemental Tables**

#### Supplemental Table 1: Study population. Laboratory Value Summary for patients indexed to start of first-LOT by race and rwOS/dRR population

|  | | **rwOS Population (N = 5809)** | | **dRR Population (N = 1824)** | |
| --- | --- | --- | --- | --- | --- |
| **Patient Characteristic, n (%)** | | **Black (N = 1172)** | **White (N = 4637)** | **Black (N = 362)** | **White (N = 1462)** |
| Hemoglobin > 8 g/dL: | Yes | 778 (66.4) | 3233 (69.7) | 276 (76.2) | 1207 (82.6) |
|  | No | 87 (7.4) | 209 (4.5) | 37 (10.2) | 80 (5.5) |
|  | Unknown | 307 (26.2) | 1195 (25.8) | 49 (13.5) | 175 (12.0) |
| Platelet count > 50,000/µL: | Yes | 807 (68.9) | 2946 (63.5) | 296 (81.8) | 1130 (77.3) |
|  | No | 19 (1.6) | 74 (1.6) | 7 (1.9) | 23 (1.6) |
|  | Unknown | 346 (29.5) | 1617 (34.9) | 59 (16.3) | 309 (21.1) |
| Absolute neutrophil count > 1.0 x 10^9^/L: | Yes | 432 (36.9) | 1618 (34.9) | 160 (44.2) | 624 (42.7) |
|  | No | 18 (1.5) | 75 (1.6) | 10 (2.8) | 27 (1.8) |
|  | Unknown | 722 (61.6) | 2944 (63.5) | 192 (53.0) | 811 (55.5) |
| Creatinine clearance > 30 mL/min: | Yes | 619 (52.8) | 2516 (54.3) | 254 (70.2) | 1044 (71.4) |
|  | No | 109 (9.3%) | 368 (7.9) | 24 (6.6) | 124 (8.5) |
|  | Unknown | 444 (37.9%) | 1753 (37.8) | 84 (23.2) | 294 (20.1) |
| Normal hepatic function: | Yes | 613 (52.3) | 2476 (53.4) | 234 (64.6) | 1021 (69.8) |
|  | No | 24 (2.0) | 80 (1.7) | 8 (2.2) | 33 (2.3) |
|  | Unknown | 535 (45.6) | 2081 (44.9) | 120 (33.1) | 408 (27.9) |

1. Lab values are determined using records up to 30 days prior to and including the 1L start date. The closest record to the 1L start date is used. For patients with multiple values recorded on the same day, the highest value is used.

####

#### Supplemental Table 2: Study population. Baseline characteristics and laboratory values of cohort indexed to start of second-LOT by race and by rwOS and dRR population

|  | | **rwOS Population (N = 3048)** | | **dRR Population (N = 572)** | |
| --- | --- | --- | --- | --- | --- |
| **Patient Characteristic** | | **Black**  **(N = 606)** | **White**  **(N = 2442)** | **Black (N = 119)** | **White (N = 453)** |
| Sex, n (%) | Female | 318 (52.5) | 1071 (43.9) | 68 (57.1) | 204 (45.0) |
|  | Male | 288 (47.5) | 1371 (56.1) | 51 (42.9) | 249 (55.0) |
| Practice Type, n (%) | Academic | 57 (9.4) | 330 (13.5) | 10 (8.4) | 44 (9.7) |
|  | Community | 549 (90.6) | 2112 (86.5) | 109 (91.6) | 409 (90.3) |
| Median age at 2L start (years) [IQR] | | 67.0 [59.2;75.0] | 71.0 [62.0;77.0] | 68.0 [61.5;75.5] | 72.0 [65.0;78.0] |
| Age at 2L start,  n (%) | <65 years | 250 (41.3) | 775 (31.7) | 42 (35.3) | 106 (23.4) |
|  | 65-74 years | 184 (30.4) | 800 (32.8) | 41 (34.5) | 172 (38.0) |
|  | 75+ years | 172 (28.4) | 867 (35.5) | 36 (30.3) | 175 (38.6) |
| Region, n (%) | Midwest | 67 (11.1) | 379 (15.5) | 13 (10.9) | 75 (16.6) |
|  | Northeast | 87 (14.4) | 526 (21.5) | 17 (14.3) | 88 (19.4) |
|  | South | 363 (59.9) | 784 (32.1) | 74 (62.2) | 174 (38.4) |
|  | West | 26 (4.3) | 374 (15.3) | 5 (4.2) | 66 (14.6) |
|  | Other/unknown | 63 (10.4) | 379 (15.5) | 10 (8.4) | 50 (11.0) |
| ECOG at 2L start, n (%)^2^ | 0-1 | 223 (36.8) | 967 (39.6) | 44 (37.0) | 201 (44.4) |
|  | >1 | 65 (10.7) | 263 (10.8) | 19 (16.0) | 56 (12.4) |
|  | Unknown | 318 (52.5) | 1212 (49.6) | 56 (47.1) | 196 (43.3) |
| ISS Stage, n (%) | Stage I | 123 (20.3) | 435 (17.8) | 26 (21.8) | 78 (17.2) |
|  | Stage II | 114 (18.8) | 487 (19.9) | 25 (21.0) | 102 (22.5) |
|  | Stage III | 97 (16.0) | 485 (19.9) | 15 (12.6) | 90 (19.9) |
|  | Unknown/not doc. | 272 (44.9) | 1035 (42.4) | 53 (44.5) | 183 (40.4) |
| Year of MM Diagnosis, n (%) | 2011 - 2013 | 225 (37.1) | 859 (35.2) | 42 (35.3) | 167 (36.9) |
|  | 2014 - 2016 | 270 (44.6) | 1117 (45.7) | 60 (50.4) | 205 (45.3) |
|  | 2017 - 2019 | 111 (18.3) | 466 (19.1) | 17 (14.3) | 81 (17.9) |
| Hemoglobin > 8 g/dL, n (%)^3^ | Yes | 479 (79.0) | 1931 (79.1) | 104 (87.4) | 403 (89.0) |
|  | No | 24 (4.0) | 66 (2.7) | 4 (3.4) | 9 (2.0) |
|  | Unknown | 103 (17.0) | 445 (18.2) | 11 (9.2) | 41 (9.1) |
| Platelet count > 50,000/µL, n (%)^3^ | Yes | 459 (75.7) | 1666 (68.2) | 102 (85.7) | 341 (75.3) |
|  | No | 16 (2.6) | 80 (3.3) | 5 (4.2) | 11 (2.4) |
|  | Unknown | 131 (21.6) | 696 (28.5) | 12 (10.1) | 101 (22.3) |
| Absolute neutrophil count > 1.0 x 10^9^/L, n (%)^3^ | Yes | 283 (46.7) | 995 (40.7) | 65 (54.6) | 203 (44.8) |
|  | No | 11 (1.8) | 77 (3.2) | 5 (4.2) | 17 (3.8) |
|  | Unknown | 312 (51.5) | 1370 (56.1) | 49 (41.2) | 233 (51.4) |
| Creatinine clearance > 30 mL/min, n (%)^3^ | Yes | 378 (62.4) | 1589 (65.1) | 89 (74.8) | 356 (78.6) |
|  | No | 61 (10.1) | 165 (6.8) | 8 (6.7) | 28 (6.2) |
|  | Unknown | 167 (27.6) | 688 (28.2) | 22 (18.5) | 69 (15.2) |
| Normal hepatic function, n (%)^3^ | Yes | 391 (64.5) | 1510 (61.8) | 96 (80.7) | 326 (72.0) |
|  | No | 12 (2.0) | 34 (1.4) | 5 (4.2) | 9 (2.0) |
|  | Unknown | 203 (33.5) | 898 (36.8) | 18 (15.1) | 118 (26.0) |
| Second-LOT, n (%) | Bort, Dex, Len | 99 (16.3) | 334 (13.7) | 16 (13.4) | 78 (17.2) |
|  | Dex,Len | 86 (14.2) | 310 (12.7) | 25 (21.0) | 60 (13.2) |
|  | Bort,Dex | 53 (8.7) | 187 (7.7) | 10 (8.4) | 42 (9.3) |
|  | Len | 38 (6.3) | 146 (6.0) | 5 (4.2) | 24 (5.3) |
|  | Bort, Cyc, Dex | 42 (6.9) | 136 (5.6) | 11 (9.2) | 22 (4.9) |
|  | Other 2L | 288 (47.5) | 1329 (54.4) | 52 (43.7) | 227 (50.1) |
| Stem Cell Transplant, n (%) | | 67 (11.1) | 222 (9.1) | 13 (10.9) | 43 (9.5) |
| Maintenance Therapy, n (%) | | 94 (15.5) | 286 (11.7) | 21 (17.6) | 55 (12.1) |
| Specimen Type for 2L Response, n (%) | FLC |  |  | 27 (22.7) | 93 (20.5) |
|  | SPEP |  |  | 92 (77.3) | 349 (77.0) |
|  | UPEP |  |  | 0 (0.0) | 11 (2.4) |
| Median follow-up time from 2L start (months) [IQR]^4^ | | 20.7 [7.7;37.3] | 18.8 [7.5;34.6] | 20.1 [8.9;34.8] | 18.8 [9.9;35.1] |
| Median time from MM Diagnosis to 2L start (months) [IQR] | | 10.3 [5.3;20.7] | 10.4 [5.7;19.2] | 15.0 [6.9;26.5] | 12.6 [6.9;25.2] |

1. Regions are based on the United States census region of the patient’s state of residence. Region is reported as Other/Unknown for patients from academic sites for de-identification reasons.
2. ECOG is determined using records from 30 days prior to and up to 7 days after the 2L start date. If there are multiple ECOG values at the same absolute distance from the 2L start date, priority is given to the ECOG value that precedes the index date. For patients with multiple ECOG values recorded on the same day, the highest value is used.
3. Lab values are determined using records up to 30 days prior to and including the 2L start date. The closest record to the 2L start date is used. For patients with multiple values recorded on the same day, the highest value is used.
4. Follow-up time is defined as time from the start date of second-line therapy to either date of death, if known, or the patient’s last confirmed activity (i.e., clinic visit or abstracted oral drug episode).

####

#### Supplemental Table 3a: Relative hazard of death from start of first line between Black patients and White patients, adjusting for Sex, Age, and Practice Type for common first-LOT regimens

|  | **aHR (95% CI)** | **Events** | **Median rwOS (95% CI), months** |
| --- | --- | --- | --- |
| **Bortezomib, Dexamethasone, Lenalidomide (N=1975)** | | |  |
| Black (N=407)  White (N=1568) | 0.95 (0.75, 1.21)  Ref. | 83  387 | 86.4 (64.6, NR)  66.2 (58.6, 75.1) |
| **Dexamethasone, Lenalidomide (N=1010)** | | |  |
| Black (N=212)  White (N=798) | 0.96 (0.74, 1.24)  Ref. | 74  325 | 73.5 (59.5, NR)  56.0 (50.2, 66.0) |
| **Bortezomib, Dexamethasone (N=849)** | | |  |
| Black (N=168)  White (N=681) | 0.94 (0.73, 1.21)  Ref. | 74  340 | 41.3 (30.4, 76.3)  36.9 (30.7, 43.3) |

White patients are the reference group.

NR: NR.

#### Supplemental Table 3b: Relative hazard of death from start of second line between Black patients and White patients, adjusting for Sex, Age, and Practice Type for common second-LOT regimens

|  | **aHR (95% CI)** | **Events** | **Median rwOS (95% CI), months** |
| --- | --- | --- | --- |
| **Bortezomib, Dexamethasone, Lenalidomide (N=433)** | | |  |
| Black (N=99)  White (N=344) | 0.85 (0.56, 1.29)  Ref. | 27  117 | 68.92 (49.28, NR)  50.66 (42.69, NR) |
| **Dexamethasone, Lenalidomide (N=396)** | | |  |
| Black (N=86)  White (N=310) | 0.65 (0.41, 1.04)  Ref. | 21  126 | NR (44.36, NR)  49.08 (42.95, 56.59) |

White patients are the reference group.

####

#### Supplemental Table 4a: Relative odds of responding to first line treatment between Black patients and White patients, adjusting for Sex, Age, and Practice Type for common first-line regimens

|  | **aOR (95% CI)** | **Responders** | **dRR (95% CI)** |
| --- | --- | --- | --- |
| **Bortezomib, Dexamethasone, Lenalidomide (N=786)** | | |  |
| Black (N=161)  White (N=625) | 0.98 (0.5, 2.06)  Ref. | 150  583 | 93.2% (88.2%, 96.1%)  93.3% (91.0%, 95.0%) |
| **Dexamethasone, Lenalidomide (N=286)** | | |  |
| Black (N=54)  White (N=232) | 1.21 (0.41, 4.42)  Ref. | 50  205 | 92.6% (82.4%, 97.1%)  88.4% (83.6%, 91.9%) |
| **Bortezomib, Dexamethasone (N=232)** | | |  |
| Black (N=45)  White (N=187) | 0.85 (0.41, 1.84)  Ref. | 32  139 | 71.1% (56.6%, 82.3%)  74.3% (67.6%, 80.1%) |

White patients are the reference group.

#### Supplemental Table 4b: Relative odds of responding to second line treatment between Black patients and White patients, adjusting for Sex, Age, and Practice Type for common second-line regimens

|  | **aOR (95% CI)** | **Responders** | **dRR (95% CI)** |
| --- | --- | --- | --- |
| **Bortezomib, Dexamethasone, Lenalidomide (N=94)** | | |  |
| Black (N=16)  White (N=78) | 2.55 (0.42, 49.15)  Ref | 15  64 | 93.8% (71.7%, 99.7%)  82.1% (72.1%, 89.0%) |
| **Dexamethasone, Lenalidomide (N=85)** | | |  |
| Black (N=25)  White (N=60) | 0.98 (0.34, 3.02)  Ref | 18  44 | 72.0% (52.4%, 85.7%)  73.3% (61.0%, 82.9%) |

White patients are the reference group.

####

### **Supplemental Figures**

#### Supplemental Figure 1: Attrition Diagram
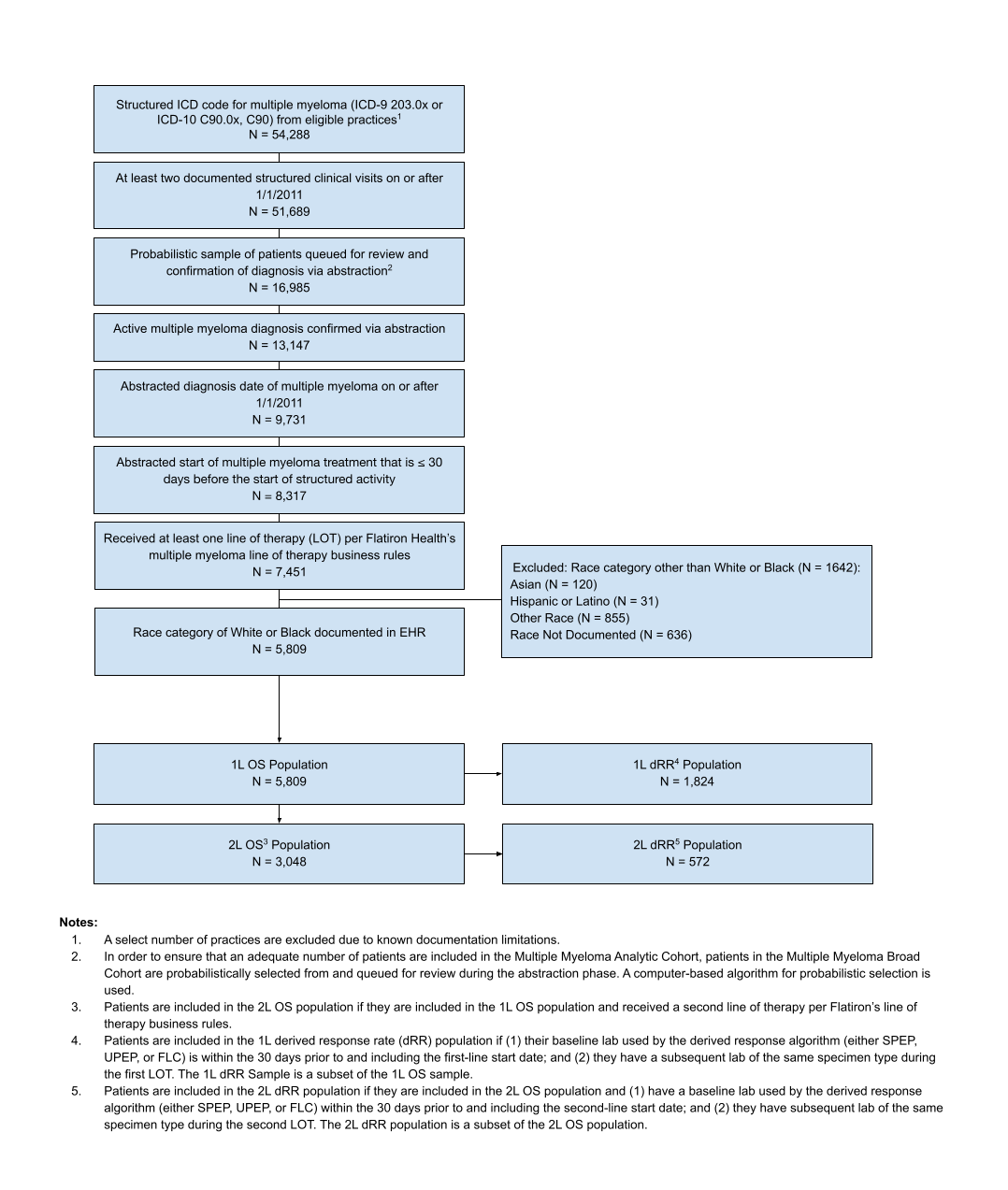

Supplement: Supplementary file 1 — Supplemental Material [file 41408_2022_665_MOESM1_ESM.docx]
